# Abusive supervision and counterproductive work behaviors under generational differences: a chain mediation model between perception of organizational politics and defensive silence

**DOI:** 10.3389/fpsyg.2025.1657730

**Published:** 2025-10-13

**Authors:** Ailin Qiu, Zhaoqi Li, MyeongCheol Choi, Hann Earl Kim

**Affiliations:** ^1^Department of Economics and Management, Neijiang Normal University, Neijiang, China; ^2^Department of Business Management, Gachon University, Seongnam, Gyeonggi, Republic of Korea

**Keywords:** abusive supervision, perceptions of organizational politics, defensive silence, counterproductive work behavior, generational differences

## Abstract

Abusive supervision leads to employees' counterproductive work behaviors, which undermines an organization's sustainable development and survival. This study posits that the relationship between abusive supervision and counterproductive work behaviors varies across different age groups. Therefore, from the perspective of generational differences (Post-80s, Post-90s, Post-00s), we explored the mediating role of perceptions of organizational politics and defensive silence on abusive supervision and counterproductive work behaviors among 441 Chinese manufacturing employees by generation. The empirical analysis was conducted using Amos and SPSS statistical software. The results showed that abusive supervision significantly positively affected counterproductive work behaviors. Additionally, it finds that perceptions of organizational politics and defensive silence each serve as mediators in the relationship between abusive supervision and counterproductive work behaviors, forming a chained mediation effect. The study showed that the results differed among the Post-80s, Post-90s, and Post-00s groups. This suggests that generational differences have different perspectives on abusive supervision and counterproductive work behaviors. These results reveal the existence of a generational gap. The main contribution of this study lies in its identification of the chain mediating effects of perceptions of organizational politics and defensive silence between abusive supervision and counterproductive work behaviors from the perspective of generational differences for the first time. This enriches the research on generational differences, abusive supervision, and counterproductive work behaviors, providing new perspectives for future research endeavors.

## Introduction

In today's multigenerational workplace, generational differences have become a frontier issue in organizational behavior research. Different cohorts—such as the Post-80s, Post-90s, and Post-00s—exhibit significant differences in values, work orientations, and communication styles. These differences not only shape employees' perceptions and reactions to leadership behaviors but may also lead to systematic variations in organizational behavior patterns (Lyons and Kuron, 2014). This dynamic is particularly salient in China's manufacturing sector, a context characterized by high power distance and hierarchical management. As a pillar of the national economy, the manufacturing industry is not only process-oriented and labor-intensive but also deeply embedded in “face culture” and authority structures (Wu and Chaturvedi, 2009). In such an environment, managers often hold highly centralized power, while employees remain in subordinate and dependent positions (Lin and Sun, 2018). At the same time, the workforce in manufacturing is composed of multiple generational groups, whose value orientations and behavioral tendencies differ markedly (Ye et al., 2020). The coexistence of hierarchical organizational structures with diverse generational expectations intensifies potential conflicts, making the manufacturing sector an ideal setting to examine how generational differences shape employees' responses to abusive supervision and their subsequent counterproductive work behaviors.

In this context, counterproductive work behavior (CWB) has attracted increasing scholarly attention. CWB is commonly defined as employees' intentional actions that harm organizations and their stakeholders (Spector et al., 2006), encompassing behaviors such as sabotage (Ambrose et al., 2002), workplace incivility (Andersson and Pearson, 1999), and retaliation (Bies and Tripp, 2005). Subsequent research has expanded this construct to include bullying (LaVan and Martin, 2008), cyberloafing (Blanchard and Henle, 2008; Blau et al., 2006), and counter-citizenship behaviors (Lee and Allen, 2002), and has more broadly conceptualized it as deliberate engagement in unethical, unlawful, or otherwise undesirable actions (Sulea et al., 2013). CWB not only reduces productivity and increases turnover but also undermines customer satisfaction and profit margins (Carpenter et al., 2020). Moreover, it fosters the spread of distrust and negativity within organizations, further eroding the work climate and employee wellbeing (Rehman and Shahnawaz, 2018). Therefore, reducing CWB and fostering a positive, trust-based work environment has become a critical issue in organizational management.

A growing body of research has consistently identified abusive supervision as a critical antecedent of CWB. Tepper (2000) not only provided the seminal definition of abusive supervision but also demonstrated its significant effect on increasing subordinates' hostility and deviant behaviors. Subsequent studies further confirmed that abusive supervision induces workplace deviance and retaliatory behaviors through negative reciprocity beliefs (Mitchell and Ambrose, 2007), and these findings have been replicated across different cultural and industrial contexts (Aryee et al., 2007). At the same time, other research has shown that this destructive leadership behavior also undermines employees' emotional experiences (Pircher Verdorfer et al., 2024), weakens the positive effects of organizational recognition and decision-making authority on mental health, thereby increasing turnover intentions (Li and Song, 2024), and deteriorates team communication, intensifies conflicts, and reduces overall performance (Lin et al., 2022; Tziner et al., 2023). Taken together, these findings suggest that abusive supervision not only directly triggers CWB but also damages individual wellbeing and organizational sustainability through multiple pathways.

The relationship between abusive supervision and CWB does not occur directly but rather evolves progressively through employees' cognitive and behavioral responses under conditions of resource threat. Conservation of Resources (COR) theory (Hobfoll, 1989) posits that when individuals face resource threats or losses, they adopt a series of cognitive and behavioral strategies to protect and conserve their remaining resources. Within this framework, perceptions of organizational politics (POP) can be understood as a negative cognitive appraisal, whereby employees construe the organizational environment as one characterized by unfairness and manipulative power dynamics (Randall et al., 1999). Such perceptions not only undermine employees' trust, satisfaction, and performance (Ellen et al., 2022; Palmer et al., 2020; Castanheira et al., 2022), but also suppress voice behavior in the absence of psychological safety (Ullah et al., 2023) and induce knowledge hiding and diminished creativity (Malik et al., 2019). From a COR perspective, POP functions as a “cognitive alarm” of resource depletion, signaling that resources are being—or are likely to be—eroded.

Building on this cognitive appraisal, employees often adopt defensive silence as a behavioral coping strategy. Defensive silence represents a typical avoidance-oriented form of resource preservation, whereby employees refrain from expressing opinions or sharing information in order to avoid potential negative consequences (Morrison and Milliken, 2000; Dyne et al., 2003). While this strategy may temporarily reduce the additional resource expenditure associated with speaking up, in the long term it undermines constructive team communication and collaboration (Liang et al., 2012), exacerbates negative emotions and organizational disengagement, and ultimately propels employees toward CWB. In other words, POP triggers cognitive perceptions of resource loss, while defensive silence embodies behavioral avoidance responses. Together, they constitute a chain mediation mechanism through which abusive supervision is gradually transformed into employees' counterproductive behaviors.

Although the above logic provides a theoretical foundation for explaining how abusive supervision influences CWB through cognitive and behavioral pathways, existing research still has notable limitations. First, the moderating role of generational differences has been largely neglected. Early studies generally assumed homogeneity in employees' responses to abusive supervision (Priesemuth and Schminke, 2019), without fully considering the boundary conditions shaped by differences in socialization experiences and digital nativity across generations. Although research on generational differences in the Chinese context has been increasing (Zhao et al., 2024), systematic comparisons among cohorts such as the Post-80s, Post-90s, and Post-00s remain insufficient against the backdrop of rapidly changing socio-cultural conditions. Second, existing studies on mediating mechanisms are overly simplified, lacking integrated exploration of chain mediation pathways that encompass both cognitive appraisal and behavioral coping. Particularly in China's high power-distance and “face culture” context, the sequential mediation mechanism of “POP → defensive silence → CWB” has yet to be sufficiently validated empirically. Third, empirical evidence relies heavily on Western or service-industry samples, with limited attention to the unique organizational structures, management models, and generational composition of China's manufacturing sector. As a pillar industry of the national economy, China's manufacturing sector is characterized by process-oriented work, high labor intensity, and hierarchical management deeply embedded in a high power-distance culture. In this context, managers often hold highly centralized authority, while employees occupy subordinate positions and lack institutionalized channels to address unfair treatment. Consequently, manufacturing employees are not only more vulnerable to the negative impacts of abusive supervision but also more likely to translate such experiences into CWB through cognitive appraisal and behavioral coping.

To address these gaps, this study drawing on COR theory, constructs a mediation model to examine how abusive supervision can lead to counterproductive work behaviors through POP and defensive silence, under the condition of generational differences (Post-80s, Post-90s, Post-00s). This model not only contributes to a deeper understanding of the impact mechanisms of abusive supervision but also provides a possible intervention strategy for organizations to mitigate the negative impacts of abusive supervision, improve the work environment, and reduce the occurrence of counterproductive work behaviors. Meanwhile, this study adds depth and richness to the literature through the generational differences perspective and provides managers with targeted organizational resources based on the different age groups of employees.

## Theory and hypotheses

### Theory of generations

The theory of generational differences, first proposed by the German sociologist Mannheim (1952), suggests that a generation or generation cohort is a group of people who share a common location in social and historical processes, and who have similar experiences that lead to convergent patterns of thinking and behavior. Based on this perspective, generations are recognized as identifiable groups of people who share the same birth year and have gone through key stages of growth together (Kupperschmidt, 2000). Differences between generations are not only in terms of age but also in terms of value differences between generational groups, which are expressed in terms of differences in work perceptions, value judgments, and behavioral styles (Dencker et al., 2008).

To study generational differences, it is necessary to first clarify how generational groups are divided. Western scholars, such as those in the United States, have proposed division into four categories. Baby Boomers were born between 1946 and 1964 (Swanzen, 2018; Witcher and Sasso, 2024). Generation X consists of individuals born between 1965 and 1980 (Swanzen, 2018). Generation Y refers to the millennials born between 1980 and 1994 (Berkup, 2014). Generation Z comprises those born between 1995 and 2012 (Pichler et al., 2021). Chinese scholars categorize generational cohorts based on birth years, dividing them into 10-year cycles, while aligning the categorization with significant historical events in China that have shaped the social environment. In this study, we consider Chinese employees as the research object and refer to Shi and Guo (2023) delineation. The 1950s generation (the “Post-50s”) represents the generation at the beginning of the People's Republic of China. The 1960s generation (the “Post-60s”) represents the generation of socialist construction, and the 1970s generation (the “Post-70s”) represents the generation of reform and opening-up. The 1980s generation (the “Post-80s”) represents the generation of socialist construction and family planning policy. The 1990s generation (the “Post-90s”) represents the generation of globalization and informationization (Shi and Guo, 2023). The 2000s (the “Post-00s”) are also known as the “WTO Generation” and the “Power Generation” because of China's accession to the World Trade Organization in 2001.

There are significant cognitive and social differences between generations, which result in the development of different values, leading to changes in mental attitudes and behavior (Dam et al., 2023; Schullery, 2013; Sharabi et al., 2019; Torsello, 2019; Mahmoud et al., 2024).

### Abusive supervision and counterproductive work behaviors

Abusive supervision has been shown to elicit a wide range of negative emotions in employees, including anger, frustration, and helplessness (Tepper, 2000). Specifically, abusive supervision not only provokes anger and anxiety but also diminishes positive emotions, thereby undermining employees' life satisfaction and work engagement (Santos et al., 2023; Yu et al., 2023; Gip et al., 2024). Such negative experiences often lead to psychological exhaustion and persistent rumination over adverse events, resulting in psychological imbalance and reduced wellbeing (Asim et al., 2023).

From the perspective of COR theory, abusive supervision constitutes a severe resource-threatening condition that erodes employees' psychological resources and social support (Hobfoll, 1989). As resources are continuously depleted, employees are more likely to adopt defensive or retaliatory strategies to conserve or restore their remaining resources, with CWB representing a typical maladaptive coping response (Akram et al., 2019; Gallegos et al., 2021). Prior studies have further demonstrated that employees with lower emotional stability are particularly vulnerable to abusive supervision, exhibiting higher levels of CWB (Spector and Fox, 2002). In addition, abusive leadership has been found to diminish employees' autonomous motivation, reduce job satisfaction and innovative behavior, and consequently increase CWB (Ronen and Donia, 2020). Notably, research has also indicated that subordinates' counterproductive behaviors may themselves act as antecedents of abusive supervision, creating a reciprocal cycle (Abdullah and Al-Abrrow, 2025). Overall, supervisors' abusive behaviors trigger negative emotional reactions among employees, thereby heightening the likelihood of CWB in both interpersonal and organizational domains (Junça Silva and Encarnação, 2024).

*H1. Abuse of supervision is positively related to counterproductive work behaviors*.
*H2. The effectiveness of abusive supervision on counterproductive work behaviors is influenced by generational differences*.

### The mediating role of perceptions of organizational politics

According to COR theory, when employees encounter resource threats, they develop cognitive defense mechanisms to assess and interpret situational stressors (Hobfoll et al., 2018; Pandey et al., 2021). Under abusive supervision, employees are more likely to perceive intensified power struggles and unfair resource allocation within the organization, leading to negative perceptions of organizational politics (Ozer et al., 2024). Such negative appraisals not only undermine employees' trust and sense of belonging but also increase their likelihood of engaging in CWB (De Clercq and Pereira, 2024).

Furthermore, abusive supervision aggravates employees' perceptions of injustice and political manipulation, reinforcing their sense of alienation and prompting deviant behaviors (Vigoda-Gadot and Talmud, 2010). Empirical evidence shows that Machiavellian leadership promotes CWB through abusive supervision, and this effect is even stronger when leaders display higher levels of political behavior (Cai et al., 2024). In high-POP contexts, employees often feel betrayed by the organization and respond through behaviors such as performance reduction, absenteeism, or withdrawal (Makhdoom et al., 2019). POP has also been shown to negatively correlate with employee performance, while increasing job stress and CWB (Al-Romeedy and Khairy, 2024).

From the COR perspective, POP acts as a “cognitive alarm” of resource loss, reflecting employees' heightened sensitivity to unfairness and manipulation in the organizational environment, which subsequently drives CWB (Zivnuska et al., 2004; De Clercq and Pereira, 2024). Particularly in high-POP situations, employees experience greater concerns about job security and career advancement, making them more likely to engage in CWB as a maladaptive form of resource protection or compensation (Son et al., 2023; Al-Romeedy and Khairy, 2024).

*H3. Perceptions of organizational politics mediate the relationship between abusive supervision and counterproductive work behaviors*.
*H4. The mediation effect of perceptions of organizational politics between abusive supervision and counterproductive work behaviors is influenced by generational differences*.

### The mediating role of defensive silence

According to COR theory, defensive silence is a behavioral coping strategy adopted under conditions of resource scarcity or threat, through which employees refrain from speaking up to avoid further resource loss (Hobfoll, 1989). When confronted with abusive supervision, employees often reduce social exchange and choose to remain silent in order to minimize the depletion of emotional resources (Cheng et al., 2023). This silence is manifested not only in the lack of feedback toward supervisors but also in withholding opinions from colleagues and the organization as a whole (Dyne et al., 2003). Prior research has shown that abusive supervision undermines employees' sense of interactional justice, thereby reducing their willingness to express opinions (Wang and Jiang, 2015). Moreover, when employees feel undervalued by their leaders, they are more likely to engage in silence (Hamstra et al., 2021).

Although defensive silence may seem to help employees “stop the loss” in the short term by avoiding additional resource expenditure, in the long run it weakens constructive organizational interaction and social exchange, leading to the accumulation of negative emotions and feelings of disengagement. This, in turn, significantly increases the likelihood of employees engaging in CWB (Omran and Samar Altayeb Ahmed Kamal, 2021). For instance, defensive silence undermines organizational norms and performance through knowledge hiding, thereby triggering CWB (Qi and Ramayah, 2022).

Furthermore, destructive leadership often fosters a climate of silence by reinforcing compulsory organizational citizenship behaviors and sustained commitment (Tahir et al., 2025). In such contexts, employees, concerned about power suppression, are more likely to feel a loss of autonomy and helplessness, which drives them to adopt defensive silence (Millender et al., 2023). At the same time, leader ostracism threatens employees' efficacy needs, pushing them into defensive silence, which subsequently leads to emotional exhaustion, declines in trust, morale, and motivation, and ultimately culminates in burnout and CWB (Jahanzeb et al., 2018).

*H5. Defensive silence mediates the relationship between abusive supervision and counterproductive work behaviors*.
*H6. The mediation effect of defensive silence between abusive supervision and counterproductive work behaviors is influenced by generational differences*.

### Defensive silence and the chain mediation of perceptions of organizational politics

Abusive supervision represents a prototypical form of intense resource threat, continuously depleting employees' psychological resources (e.g., self-esteem, emotional stability) and social support resources (e.g., trust, leader–subordinate relationships; Hobfoll, 1989). Under such high-pressure conditions, employees are likely to develop both cognitive and behavioral defensive mechanisms. These mechanisms are not isolated reactions; rather, they interact and unfold progressively, giving rise to what COR theory describes as a “resource loss spiral.”

Specifically, abusive supervision first activates employees' POP. In this stage, employees interpret the organizational environment as one characterized by unfairness and manipulative power dynamics (Randall et al., 1999), which heightens concerns about potential resource depletion. This cognitive defensive response not only undermines employees' trust, satisfaction, and work engagement (Ellen et al., 2022), but also generates additional psychological strain and insecurity, thereby intensifying the perception of resource loss.

Building on this appraisal, employees frequently adopt defensive silence as a behavioral coping strategy. Defensive silence is a typical avoidance-oriented form of resource preservation, whereby employees deliberately withhold opinions or information to avoid conflict, retaliation, or further resource depletion (Dyne et al., 2003). While such avoidance may provide short-term “damage control,” it simultaneously cuts off important channels for resource replenishment, such as leader support, coworker assistance, and organizational feedback. Over time, this erosion of constructive interaction and social exchange exacerbates emotional exhaustion and organizational disengagement (Liang et al., 2012).

Ultimately, this sequential process—triggered by abusive supervision and unfolding through cognitive (POP) and behavioral (defensive silence) defenses—culminates in CWB. Prolonged exposure to perceptions of unfairness, silence, and social isolation increases the likelihood that employees will engage in retaliatory or opportunistic behaviors, such as withdrawal, sabotage, deception, or knowledge hiding, as maladaptive means of expressing dissatisfaction or compensating for resource loss (Omran and Samar Altayeb Ahmed Kamal, 2021). These behaviors not only accelerate the deterioration of employee–organization relationships but also further amplify resource depletion, thereby locking employees into a vicious cycle.

In sum, from the perspective of COR theory, abusive supervision does not directly lead to CWB in a linear fashion. Instead, it operates through the chain mediation pathway of POP (cognitive defense) → defensive silence (behavioral defense) → CWB (externalized outcome of resource loss). This pathway reflects COR's core logic of a “resource loss spiral,” whereby initial resource threats trigger cognitive and behavioral reactions that progressively magnify resource depletion and ultimately manifest in systemic negative outcomes.

**Figure 1:**
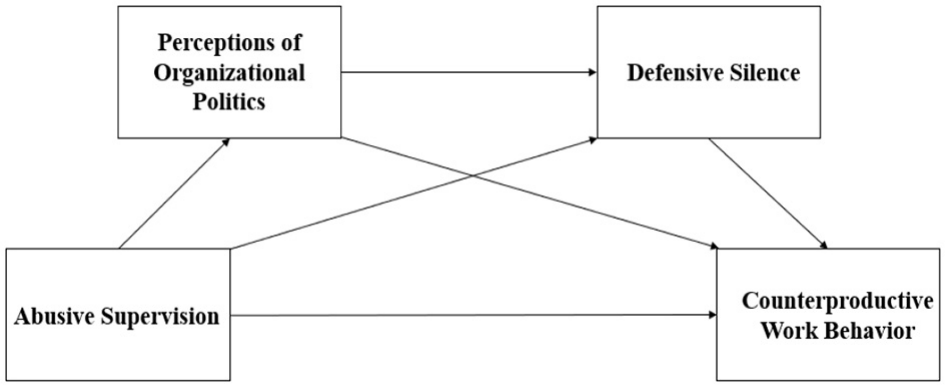
Research model.

*H7. Perceptions of organizational politics and defensive silence mediate the relationship between abusive supervision and counterproductive work behaviors in a chained mediation model*.
*H8. Perceptions of organizational politics and defensive silence mediate the relationship between abusive supervision and counterproductive work behaviors in a chained mediation model is influenced by generational differences*.
Based on the above theoretical hypotheses, this study constructs a moderated mediation model (see Figure 1).

## Methods

### Sample and data collection

Survey data were collected from employees residing in the Jiangsu, Guangdong, Sichuan, and Shandong provinces of China. Data were collected using electronic questionnaires. The employees were primarily from the manufacturing sector. The three generational groups of survey participants included: the Post-00s group (under 25 years), the Post-90s group (25–35 years), and the Post-80s group (36–45 years).

To ensure a balanced representation of the three generational groups, questionnaires were distributed to local universities, research institutes, and specialized survey agencies, who were entrusted with handling the distribution process and keeping the number of participants from each generational group approximately equal. Random sampling was then performed. To ensure the authenticity and validity of the data, emotionally charged language was avoided, the names of relevant variables were hidden, and questions were designed to avoid social desirability bias. Additionally, the purpose of this survey was explained to the participants and they were assured that their responses would remain confidential to alleviate any concerns. Finally, responses were received from a diverse and representative sample of participants. Of the 540 questionnaires, 441 valid responses were received, for a response rate of 81.7%. To achieve a relatively balanced sample size between the groups for better comparisons, we randomly selected a sample of 160 participants from each group based on generational differences. From these participants, we further collected a valid sample of participants, and finally came up with 151 participants from the Post-80s group, 156 participants from the Post-90s group, and 134 participants from the Post-00s group, for a total of 441 participants (the full-sample group). Participants' demographic characteristics are listed in Table 1, the names of relevant variables were hidden, and questions were designed to avoid social desirability bias. Additionally, the purpose of this survey was explained to the participants and they were assured that their responses would remain confidential to alleviate any concerns. Finally, responses were received from a diverse and representative sample of participants. Of the 540 questionnaires, 441 valid responses were received, for a response rate of 81.7%. To achieve a relatively balanced sample size between the groups for better comparisons, we randomly selected a sample of 160 participants from each group based on generational differences. From these participants, we further collected a valid sample of participants, and finally came up with 151 participants from the Post-80s group, 156 participants from the Post-90s group, and 134 participants from the Post-00s group, for a total of 441 participants (the full-sample group). Participants' demographic characteristics are listed in Table 1.

**Table 1:** Demographic analysis.

**Demographic variable**	**Type**	**Post-80s group (*****N*** = **151)**		**Post-90s group (*****N*** = **156)**		**Post-00s group (*****N*** = **134)**	
		**Frequency**	**%**	**Frequency**	**%**	**Frequency**	**%**
Gender	Male	78	51.66	79	50.64	69	51.49
	Female	73	48.34	77	49.36	65	48.51
Educational level	High school and under	11	7.28	16	10.26	13	9.7
	College and undergraduate	103	68.21	108	69.23	114	85.08
	Master's and above	37	24.51	32	20.51	7	5.22
Tenure	Less than 5 years	19	12.58	75	48.08	132	98.51
	6–10 years	22	14.57	53	33.97	2	1.49
	11–15 years	78	51.66	25	16.03	0	0
	16–20 years	26	17.22	2	1.28	0	0
	More than 20 years	6	3.97	1	0.64	0	0

### Measures

The measurement scales used in this study were derived from scales with known validity and reliability. A double-translation process was conducted to modify all scales to ensure accuracy. Participants were asked to rate questionnaire items (statements) on a Five-point Likert-type scale ranging from 1 (strongly disagree) to 5 (strongly agree).

Abusive supervision was assessed using the scale developed by Tepper (2000), which included six-item. The items included: “My boss told me that my thoughts and feelings were stupid.”

Perceptions of organizational politics were adopted from a scale developed by Kacmar and Carlson (1997), 15-item were used in this study, and items included: “Promotions around here are not valued much because how they are determined is so political.”

Defensive silence was assessed using a five-item scale developed by Dyne et al. (2003). The items included: “I do not offer solutions to problems because I am dominated by fear.”

Counterproductive work behavior was assessed using the eight-item scale proposed by Yang and Diefendorff (2009). The items included: “Intentionally worked slower than you could have worked.”

## Results

### Common method bias

We conducted Harman's one-way test to explore the possibility of common method bias. After performing a rotated factor analysis on all items in the questionnaire, a significant common method bias was indicated if only one.

### Descriptive analysis, reliability, validity, and correlations

Amos 26.0 and SPSS 25.0 software were used for analysis. As Table 2 shows, the reliability of the constructs for each group was greater than 0.8, the composite reliability (CR) value was greater than 0.7, and the average variance extracted (AVE) was greater than 0.5. Additionally, for each variable, the square root of its AVE was higher than the correlation between itself and the other variables for all datasets. The tests indicate that the correlation coefficients for the full sample, Post-80s, Post-90s, and Post-00s cohorts are all below 0.8, demonstrating no multicollinearity issues.

**Table 2:** Descriptive analysis, correlations, and discriminant validity.

**Sample**	**Variables**	**Mean**	**SD**	**Cronbach' s α**	**CR**	**AVE**	**1**	**2**	**3**	**4**
Full-sample group (*N* = 441)	ABS	2.754	1.011	0.881	0.881	0.553	(0.744)			
	POP	3.119	0.796	0.938	0.938	0.503	0.545	(0.709)		
	DS	3.037	0.895	0.865	0.865	0.562	0.533	0.676	(0.750)	
	CWB	2.780	0.978	0.905	0.905	0.544	0.687	0.561	0.611	(0.737)
Post-80s group (*N* = 151)	ABS	2.737	1.025	0.886	0.887	0.569	(0.755)			
	POP	3.135	0.820	0.939	0.939	0.508	0.612	(0.713)		
	DS	3.106	0.846	0.865	0.866	0.564	0.531	0.623	(0.751)	
	CWB	2.776	1.032	0.918	0.918	0.584	0.707	0.568	0.650	(0.764)
Post-90s group (*N* = 156)	ABS	2.938	1.002	0.884	0.884	0.560	(0.748)			
	POP	3.185	0.762	0.938	0.938	0.505	0.619	(0.711)		
	DS	3.082	0.890	0.864	0.867	0.568	0.574	0.677	(0.754)	
	CWB	2.828	0.942	0.901	0.902	0.534	0.674	0.607	0.614	(0.731)
Post-00s group (*N* = 134)	ABS	2.560	0.973	0.864	0.864	0.515	(0.718)			
	POP	3.023	0.803	0.937	0.938	0.504	0.368	(0.710)		
	DS	2.907	0.947	0.864	0.862	0.556	0.485	0.728	(0.746)	
	CWB	2.729	0.961	0.895	0.896	0.519	0.686	0.499	0.573	(0.720)

The italicized numbers in parentheses represent the square roots of the AVE at the end of each row. ABS, abusive supervision; POP, perceptions of organizational politics; DS, defensive silence; CWB, counterproductive work behavior.

A validated factor analysis was conducted using Amos 26.0 to further test discriminant validity, as shown in Table 3.

**Table 3:** Confirmatory factor analysis results.

**Sample**	**χ^2^**	**χ^2^/*df***	**RMSEA**	**GFI**	**CFI**	**IFI**	**TLI**
Full-sample group	1177.980	2.261	0.054	0.853	0.925	0.925	0.919
Post-80s group	860.039	1.651	0.066	0.760	0.896	0.897	0.888
Post-90s group	853.688	1.639	0.064	0.763	0.896	0.898	0.888
Post-00s group	1135.477	2.179	0.094	0.663	0.793	0.795	0.777

The results showed that the fitting indicators of the model in the full-sample group met the recommended values, and the model fit was acceptable. Meanwhile, most of the fitting indices of the other models met the recommended values, indicating that they all had a good fit and overall goodness of fit.

### Hypothesis testing

The results of the collinearity analysis show that the maximum VIF value for the main variables is 2.411. This indicates that there are no serious collinearity issues, and the data's analysis results are reliable. A hierarchical regression analysis was used to test the hypotheses. We first tested the full-sample group to test hypothesis. Next, we tested the three subgroups of the sample individually to analyze the effect of generational differences. The results are summarized in Table 4.

**Table 4:** Hierarchical regression analysis.

**Sample**	**Variables**	**OPP**	**DS**		**CWB**			
		**Model 1**	**Model 2**	**Model 3**	**Model 4**	**Model 5**	**Model 6**	**Model 7**
Full-sample group	Gender	0.003	−0.022	−0.024	−0.055	−0.055	−0.047	−0.049
	Edu	0.100^*^	0.034	−0.021	0.059	0.033	0.047	0.039
	Tenure	0.084	0.066	0.020	0.029	0.008	0.007	0.002
	ABS	0.550^***^	0.531^***^	0.228	0.687^***^	0.544^***^	0.506^***^	0.479^***^
	POP			0.551		0.260^***^		0.102^*^
	DS						0.341^***^	0.286^***^
	*R* ^2^	0.309	0.289	0.499	0.479	0.526	0.562	0.576
	*F*	38.882^***^	35.356^***^	71.977^***^	80.099^***^	80.306^**^	92.818^***^	81.046^***^
Post-80s group	Gender	−0.071	−0.096	−0.063	−0.116	−0.1	−0.095	−0.077
	Edu	0.033	−0.127	−0.142^*^	−0.038	−0.045	−0.015	0.007
	Tenure	0.159^*^	0.064	−0.011	0.016	−0.019	0.027	−0.015
	ABS	0.621^***^	0.526^***^	0.235^**^	0.683^***^	0.545^***^	0.198^*^	0.459^***^
	POP			0.469^***^		0.222^**^		0.05
	DS						0.482^***^	0.367^***^
	*R* ^2^	0.413	0.317	0.446	0.513	0.542	0.489	0.616
	*F*	20.413^***^	13.446^***^	19.310^***^	30.503^***^	28.347^***^	22.936^***^	23.814^***^
Post-90s group	Gender	0.011	0.004	−0.002	0.017	0.014	0.016	0.014
	Edu	0.099	0.129	0.077	0.147^*^	0.119^*^	0.105	0.100
	Tenure	−0.069	0.015	0.052	0.002	0.022	−0.003	0.009
	ABS	0.591^***^	0.591^***^	0.280^***^	0.668^***^	0.501^***^	0.480^***^	0.432^***^
	POP			0.526^***^		0.281^***^		0.152
	DS						0.318^***^	0.247^**^
	*R* ^2^	0.413	0.346	0.508	0.481	0.528	0.548	0.558
	*F*	21.119^***^	15.864^***^	25.674^***^	27.839^***^	27.769^**^	30.066^***^	26.674^***^
Post-00s group	Gender	0.050	−0.027	−0.06	−0.074	−0.089	−0.066	−0.078
	Edu	0.169^*^	0.039	−0.073	0.086	0.036	0.074	0.049
	Tenure	−0.052	0.044	0.079	0.059	0.075	0.045	0.06
	ABS	0.349^***^	0.479^***^	0.247^***^	0.663^***^	0.559^***^	0.516^**^	0.515^***^
	POP			0.664^***^		0.297^***^		0.178^*^
	DS						0.307^***^	0.179
	*R* ^2^	0.178	0.240	0.603	0.487	0.560	0.559	0.573
	*F*	5.552^***^	8.101^***^	32.136^***^	24.339^***^	26.924^***^	26.823^***^	24.112^***^

ABS, abusive supervision; POP, perceptions of organizational politics; DS, defensive silence; CWB, counterproductive work behavior. ^***^p < 0.001, ^**^ p < 0.01, ^*^p < 0.05.

We tested for main effects. As shown in Model 4, in the full-sample group, abusive supervision had a significant positive effect on counterproductive work behaviors (β = 0.687, *p* < 0.001), and H1 was supported. Similarly, a significant positive effect of abusive supervision on counterproductive work behaviors was found in the Post-80s group (β = 0.683, *p* < 0.001), the Post-90s group (β = 0.668, *p* < 0.01), and the Post-00s group (β = 0.663, *p* < 0.001), and there was a significant positive effect of abusive supervision on counterproductive work behaviors, further supporting H1. The Post-80s group (β = 0.683) had the highest validity, higher than the Post-90s group (β = 0.668), while the Post-00s group (β = 0.663) had the lowest validity. This shows a generational difference between abusive supervision and counterproductive work behaviors; therefore, H2 was supported.

To address the limitations of hierarchical regression analyses, the mediating effect between all groups was further tested using the PROCESS (Model 6) bootstrapping method with 5,000 resamples. Hypotheses 3 and 5 tested the mediating effect of POP and defensive silence. The results indicated that abusive supervision had a significant indirect effect on counterproductive work behaviors through POP (β = 0.058, 95% CI = 0.004–0.110), supporting H3. However, the results were different for the three generational groups, with the indirect effects for the Post-80s, Post-90s, and Post-00s groups (β = 0.026, 95% CI = −0.055–0.115), (β = 0.102, 95% CI = −0.034–0.217), and (β =0.057, 95% CI = −0.017–0.128), respectively. The Post-80s, Post-90s and Post-00s groups had a confidence interval containing 0, which did not serve as a mediator. So H4 was rejected.

Abusive supervision had a significant indirect effect on counterproductive work behaviors through defensive silence (β = 0.064, 95% CI = 0.036–0.097), supporting H5. However, the indirect effects of the Post-80s, Post-90s, and Post-00s groups were (β = 0.088, 95% CI = 0.025–0.156), (β = 0.060, 95% CI = 0.010–0.126), and (β =0.049, 95% CI = −0.003–0.114), respectively and the confidence intervals of the Post-00s groups contained 0, which did not play a mediating role, which led to the conclusion that the mediating role of defensive silence on abusive supervision and counterproductive work behaviors was affected by generational differences, supporting H6.

H7 tested the chain-mediating effects of POP and defensive silence. The results indicated that abusive supervision had a significant indirect effect on counterproductive work behaviors through the chain mediation of POP and defensive silence (β = 0.082, 95% CI = 0.049– 0.119). Therefore, H7 was supported. The indirect effects of the Post-80s, Post-90s, and Post-00s groups were (β = 0.107, 95% CI = 0.054–0.181), (β = 0.078, 95% CI = 0.012–0.163), and (β = 0.046, 95% CI = −0.022–0.098). However, the indirect effect of the Post-00s group did not form a chain mediation effect, which led to the conclusion that POP and defensive silencing mediated the relationship between abusive supervision and counterproductive work behaviors in the chain mediation model, which were influenced by generational differences. Thus, H8 was supported.

Finally, by comparing the data in Table 5, we found that the POP of the full-sample group accounted for 28.43% of the total indirect effects. Defensive silence of the full-sample group, Post-80s group and Post-90s group accounted for 31.37%, 39.82% and 25.10% of the total indirect effects, respectively. The full-sample, Post-80s, and Post-90s groups' POP and defensive silence of the chain mediation effect accounted for 40.20%, 48.42%, and 32.64% of the total indirect effects, respectively. Therefore, when examining individual mediating variables, the chain mediating effect of POP and defensive silence exhibited the strongest indirect influence on counterproductive work behavior across the full sample group, the post-80s group, and the post-90s group, followed by POP, with defensive silence alone demonstrating the least impact.

**Table 5:** Regression results of the mediation analysis.

**Group**	**Hypothesis**	**Effect**	**Boot SE**	**Boot LLCI**	**Boot ULCI**	**Proportion (%)**
Full-sample group	Direct effect	0.460	0.038	0.386	0.535	
	Total Indirect effect	0.204	0.030	0.143	0.263	
	H2: ABS → POP → CWB	0.058	0.027	0.004	0.110	28.43
	H3: ABS → DS → CWB	0.064	0.016	0.036	0.097	31.37
	H4: ABS → POP → DS → CWB	0.082	0.018	0.049	0.119	40.20
Post-80s group	Direct effect	0.490	0.068	0.356	0.624	
	Total Indirect effect	0.221	0.053	0.122	0.332	
	H2: ABS → POP → CWB	0.026	0.044	−0.055	0.115	
	H3: ABS → DS → CWB	0.088	0.034	0.025	0.156	39.82
	H4: ABS → POP → DS → CWB	0.107	0.033	0.054	0.181	48.42
Post-90s group	Direct effect	0.460	0.038	0.386	0.535	
	Total Indirect effect	0.239	0.064	0.110	0.360	
	H2: ABS → POP → CWB	0.102	0.059	−0.016	0.217	
	H3: ABS → DS → CWB	0.060	0.030	0.010	0.126	25.10
	H4: ABS → POP → DS → CWB	0.078	0.039	0.012	0.163	32.64
Post-00s group	Direct effect	0.529	0.066	0.394	0.531	
	Total Indirect effect	0.153	0.048	0.048	0.240	
	H2: ABS → POP → CWB	0.057	0.036	−0.017	0.128	
	H3: ABS → DS → CWB	0.049	0.029	−0.003	0.114	
	H4: ABS → POP → DS → CWB	0.046	0.025	−0.002	0.098	

The Direct effect and indirect effect estimated was tested for significance using 95% bias-corrected, bootstrapped confidence intervals. Bootstrap resampling = 5,000. ABS, abusive supervision; POP, perceptions of organizational politics; DS, defensive silence; CWB, counterproductive work behavior.

## Discussion

This study aimed to investigate the impact of abusive supervision on CWB and to examine the mediating roles of POP and defensive silence. The empirical results largely support the research hypotheses. Specifically, both POP and defensive silence were found to be significant predictors of CWB, and a chain-mediating effect was confirmed, indicating that abusive supervision indirectly influences employees' CWB through POP and defensive silence. Consistent with this, Li and Xu (2025) proposed a dual-pathway model that uncovers the complex psychological and behavioral response mechanisms through which abusive supervision influences CWB.

First, this study verifies the direct effect of abusive supervision on CWB. The results show that this positive relationship varies across generational cohorts: the effect was strongest among the Post-80s, followed by the Post-90s, and weakest among the Post-00s. A possible explanation is that most Post-00s employees are newcomers to the workplace, still in the stage of exploration and learning, and have not yet established a stable position within organizations. They tend to work hard to reciprocate their leaders' trust (Sluss and Thompson, 2012) and actively engage in learning (Bezuijen et al., 2010). As a result, even when facing abusive supervision, its negative impact on this cohort is relatively weak.

Second, POP partially mediated the relationship between abusive supervision and CWB, but the mediation effect was not significant across the generational subsamples. In the Chinese context, generational differences shape employees' sensitivity to organizational politics. Post-80s employees may be more inclined to attribute problems to individual leaders rather than to broader organizational politics; Post-90s employees often adopt a strategy of “limited tolerance plus self-regulation” to downplay perceptions of POP; while Post-00s employees are more likely to cope through open communication or job mobility. These differences may weaken the mediating effect of POP across cohorts (Lyons and Kuron, 2014). Moreover, when the total sample was divided into three subsamples, the reduction in sample size may have lowered statistical power, which could also explain the non-significant mediating effects.

Third, the mediating role of defensive silence between abusive supervision and CWB was found to differ across generations. For Post-80s and Post-90s employees, defensive silence played a significant mediating role. Having grown up in cultural contexts that emphasized authority and hierarchy, these employees are more likely to adopt endurance and silence when facing abusive supervision (Milliken and Morrison, 2003; Song et al., 2024). Compared with Post-80s and Post-90s, Post-00s employees, who grew up in a digital environment, enjoy wider access to social and informational resources. From the perspective of COR theory, they can compensate for threatened resources by leveraging online networks, peer communities, and digital platforms, rather than falling into a loss spiral. This resource substitution mechanism enables them to cope with abusive supervision through open communication or external support, instead of defensive silence. Thus, their digital nativity represents not only a generational trait but also a structural advantage in accessing compensatory resources.

Fourth, this study also examined the chain-mediating effect of POP and defensive silence between abusive supervision and CWB, revealing variations across generational cohorts. The findings show that the Post-00s did not exhibit a significant chain mediation effect. Instead, Post-00s employees are more likely to express grievances directly or seek external support rather than choosing silence. This suggests that the manifestation of the resource loss spiral differs across generations, with generational context serving as a boundary condition.

Finally, this study provides a deeper interpretation of generational differences. The Post-80s, who grew up during the early stages of China's reform and opening-up, experienced limited economic prosperity but a strong emphasis on collectivism (Lian, 2014). They tend to value career stability and the realization of personal worth while bearing heavy family and financial responsibilities (Yi et al., 2010). Consequently, when facing abusive supervision, they are less likely to quit and more inclined to endure in silence. Over time, the prolonged suppression of negative emotions may be transformed into CWB as a form of covert resistance. The Post-90s, in contrast, grew up during a period of rapid economic growth and social diversification. With higher educational attainment, they place greater emphasis on work–life balance, view work as a source of enjoyment, and pursue individuality and innovation (Wan and Liu, 2020). When faced with abusive supervision, they often adopt limited silence as a temporary coping strategy but retain multiple alternatives such as job change, transfer, or other forms of expression. Thus, while defensive silence mediates the relationship for this cohort, the effect is more flexible and weaker compared to the Post-80s. The Post-00s, born during China's era of rapid development and deep informatization, enjoy relatively favorable material conditions and greater opportunities for self-expression. Having been exposed to the Internet and social media from an early age, they display stronger individualistic tendencies and weaker group orientation (Wei and Juan, 2020). Accordingly, they are more inclined to cope with abusive supervision through communication, feedback, or job mobility rather than silence, which explains why defensive silence did not demonstrate a significant mediating effect in this cohort.

## Implications

### Theoretical implications

Abusive supervision and CWB have attracted increasing attention in recent years, yet our understanding of their relationship remains limited. This study aims to explore how abusive supervision influences CWB through the chain-mediating roles of POP and defensive silence. The findings not only enrich the literature on the outcome variables of abusive supervision and the antecedents of CWB but also further extend the application of COR theory, offering valuable theoretical insights.

First, from the perspective of COR theory, this study reveals how abusive supervision constitutes a severe resource-threatening condition that triggers both cognitive and behavioral defensive mechanisms, thereby driving employees into a “resource loss spiral.” This process is validated through the sequential pathway of “POP (cognitive defense) → defensive silence (behavioral defense) → CWB (behavioral outcome),” which concretely reflects the “resource loss chain” emphasized in COR theory. Thus, this study not only confirms the link between abusive supervision and CWB but also deepens theoretical understanding of how resource threats translate into counterproductive behaviors.

Second, this study finds that the effects of abusive supervision vary across generational groups. This discovery extends the boundary conditions of COR theory, demonstrating that resource loss processes are not uniform but are moderated by employees' developmental backgrounds and value orientations. In other words, generational differences act as a critical contextual variable that shapes how individuals cognitively and behaviorally respond to resource threats. This conclusion not only provides new evidence for generational research but also integrates COR theory with intergenerational studies, generating cross-domain theoretical contributions.

Finally, by incorporating POP and defensive silence as chain-mediating variables in the abusive supervision–CWB relationship, this study addresses the limitation of prior research that has tended to oversimplify mediating mechanisms. By combining cognitive appraisal and behavioral coping, this study presents a more comprehensive account of the “cognition–behavior–outcome” dynamic emphasized in COR theory. This finding offers a richer theoretical framework for subsequent research and enhances understanding of how destructive leadership translates into employees' negative behavioral outcomes through multi-level resource loss pathways.

### Practical implications

The findings of this study have important practical implications for managers. First, abusive supervision is often covert and progressive. In high power-distance contexts, employees may choose tolerance over resistance, making early detection difficult. Organizations should therefore establish monitoring and early-warning systems, such as 360-degree feedback, anonymous surveys, and grievance channels, supported by third-party arbitration mechanisms to ensure employees can report issues safely. Regular leadership training and practical case learning should also be conducted to raise managers' awareness of the long-term harm of abusive supervision and to prevent its recurrence.

Second, in China's manufacturing sector, managers often rely on authority and control, which can escalate into abusive supervision. To counter this, organizations should promote servant and empowering leadership, strengthen trust and support, and introduce coaching-oriented management to replace top-down control. Leadership behaviors should also be incorporated into performance evaluations, emphasizing respect, fairness, and the creation of a positive climate.

Third, generational cohorts vary in values and work attitudes, requiring tailored approaches. Post-80s employees, who emphasize stability and family responsibilities, need clear career pathways, fair promotions, and security mechanisms. Post-90s employees, who value work–life balance and innovation, benefit from flexible work arrangements and autonomy-supportive practices. Post-00s employees, who emphasize self-expression and open communication, require multi-channel feedback platforms and inclusive cultures that encourage positive interaction over silence or withdrawal.

Fourth, psychological safety is essential to buffer the negative effects of abusive supervision. Organizations should enforce zero-tolerance policies against retaliation, build cultures where mistakes and dissenting opinions are acceptable, and encourage leaders to actively solicit and respond to employee input. This is especially critical for younger employees, who value openness and expression. A climate of psychological safety not only reduces defensive silence but also promotes engagement, innovation, and overall performance.

## Limitations and future research

Although this study provides valuable insights, it also has several limitations. First, the focus on three generational cohorts (Post-80s, Post-90s, and Post-00s) narrows the generalizability of the findings, and subgroup analyses reduced sample sizes, lowering statistical power and contributing to some non-significant effects. Future research should broaden generational coverage and ensure larger, balanced samples. Second, data were collected mainly from China's manufacturing sector. While this context provides a valuable setting, it may limit the applicability of results to other industries or cultural contexts. Future studies should extend to diverse industries and cultures to improve external validity. Third, the cross-sectional quantitative design cannot fully capture the dynamic evolution of abusive supervision and counterproductive work behaviors. Longitudinal or mixed-method approaches would yield richer insights. Fourth, the model focused only on perceptions of organizational politics and defensive silence, while other factors such as personality traits, work experience, organizational structures, or cultural influences may also play critical roles. Future research should incorporate additional mediators and moderators to build a more comprehensive framework.

## Data Availability

The original contributions presented in the study are included in the article/supplementary material, further inquiries can be directed to the corresponding authors.
